# Supplementary material for: Modeling glioblastoma heterogeneity as a dynamic network of cell states
Source: Mol Syst Biol. 2021 Sep 16;17(9):e10105. doi: 10.15252/msb.202010105 (PMC8444284; doi:10.15252/msb.202010105)
Supplement: Supplementary file 6 — Source Data for Figure 5 [file MSB-17-e10105-s004.zip › Figure5A_sourcedata/GSEA_3017/hallmarks_stateD.GseaPreranked.1621934542601/HALLMARK_ANDROGEN_RESPONSE.html]

Details for gene set HALLMARK\_ANDROGEN\_RESPONSE[GSEA]

|  || Dataset | state23017 |
| Phenotype | NoPhenotypeAvailable |
| Upregulated in class | na\_pos |
| GeneSet | HALLMARK\_ANDROGEN\_RESPONSE |
| Enrichment Score (ES) | 0.16180976 |
| Normalized Enrichment Score (NES) | 0.777065 |
| Nominal p-value | 0.7752809 |
| FDR q-value | 0.7686871 |
| FWER p-Value | 0.959 |
Table: GSEA Results Summary

  

Fig 1: Enrichment plot: HALLMARK\_ANDROGEN\_RESPONSE      
 Profile of the Running ES Score & Positions of GeneSet Members on the Rank Ordered List

  

| PROBE | GENE SYMBOL | GENE\_TITLE | RANK IN GENE LIST | RANK METRIC SCORE | RUNNING ES | CORE ENRICHMENT || 1 | IDI1 |  |  | 34 | 0.382 | 0.0385 | Yes |
| 2 | SMS |  |  | 45 | 0.358 | 0.0913 | Yes |
| 3 | DBI |  |  | 57 | 0.339 | 0.1402 | Yes |
| 4 | HMGCR |  |  | 141 | 0.262 | 0.1208 | Yes |
| 5 | INSIG1 |  |  | 150 | 0.258 | 0.1583 | Yes |
| 6 | ITGAV |  |  | 217 | -0.255 | 0.1508 | Yes |
| 7 | HOMER2 |  |  | 446 | -0.279 | 0.0232 | Yes |
| 8 | CDK6 |  |  | 538 | -0.290 | 0.0023 | Yes |
| 9 | ACTN1 |  |  | 729 | -0.324 | -0.0886 | Yes |
| 10 | LIFR |  |  | 755 | -0.333 | -0.0516 | Yes |
| 11 | STK39 |  |  | 858 | -0.359 | -0.0692 | Yes |
| 12 | PMEPA1 |  |  | 868 | -0.363 | -0.0148 | Yes |
| 13 | PIAS1 |  |  | 901 | -0.375 | 0.0241 | Yes |
| 14 | PDLIM5 |  |  | 941 | -0.390 | 0.0600 | Yes |
| 15 | AKAP12 |  |  | 994 | -0.412 | 0.0898 | Yes |
| 16 | ARID5B |  |  | 1095 | -0.467 | 0.0919 | Yes |
| 17 | SAT1 |  |  | 1109 | -0.473 | 0.1618 | Yes |
Table: GSEA details [plain text format]

  

Fig 2: HALLMARK\_ANDROGEN\_RESPONSE: Random ES distribution      
 Gene set null distribution of ES for **HALLMARK\_ANDROGEN\_RESPONSE**
